# Voluntary assisted dying: challenges in Northern Territory remote Aboriginal communities

**DOI:** 10.5694/mja2.70023

**Published:** 2025-08-14

**Authors:** Geetanjali Lamba, Kane Vellar, C Paul Burgess, Camille La Brooy, Paul A Komesaroff

**Affiliations:** ^1^ Monash University Melbourne VIC; ^2^ Northern Territory Department of Health Darwin NT; ^3^ Monash Bioethics Centre Monash University Prahran VIC; ^4^ Melbourne University Melbourne VIC

**Keywords:** Health policy, Rural health services, Population health, Population policy, Public policy, Euthanasia, Legislation, medical, Terminal care‐key

Voluntary assisted dying (VAD) legislation has now been passed in all Australian jurisdictions, except for the Northern Territory.^1^, ^2^ The Voluntary Assisted Dying Independent Expert Advisory Panel led public consultations in the NT to inform development of NT VAD legislation, submitting their report to the Chief Minister in July 2024, which has been publicly released.^3^ This perspective article reflects on the VAD implementation challenges highlighted in this public report. We discuss the unique demographics of the NT, including a significant Aboriginal population living in remote areas coupled with a high burden of chronic disease, which poses difficulties for equitable access to end‐of‐life services (respecting NT cultural protocols, “First Nations” in this perspective article are referred to as “Aboriginal peoples”, which is inclusive of Torres Strait Islanders).^3^ “Cultural safety” is critical for Aboriginal peoples but application is contextual. Telehealth, which may help increase access, also presents challenges.

## Northern Territory context

About 30% of the NT population identifies as Aboriginal, with 75% of NT Aboriginal peoples living in remote or very remote areas, compared with 15.4% nationally.^4^ In contrast, 24% of non‐Indigenous people in the NT live in remote or very remote areas.^5^, ^6^ The NT has a 77% higher burden of disease than the national average, with NT Aboriginal peoples experiencing a disproportionate disease burden that is 3.6 times higher than that of non‐Indigenous people in the NT.^7^ Remoteness is also associated with reduced access to health care, potentially including VAD services.

The Northern Territory Government health department, NT Health, manages six public hospitals and 39 remote primary health care (PHC) centres, and supports 133 clinics or services operated by Aboriginal Controlled Community Health Organisations.^8^ In addition to vast distances, the health system is beleaguered by workforce shortages, a high workforce turnover, cross‐cultural challenges, and profound socio‐economic disadvantage.^9^, ^10^ Thus, equitable access to culturally safe health services, particularly for palliative and end‐of‐life care, including VAD, will require both policy and resource commitments.

## Legal context

The NT was the first Australian jurisdiction to legalise VAD in 1995 before losing this right under a federal government in 1997. The Restoring Territory Rights Bill 2022^11^ lifted this restriction.

The Commonwealth *Criminal Code Act 1995* prohibits the use of carrier services (telephone, email or internet communications) to disseminate suicide‐related materials.^12^ Significant legal barriers remain in the use of telehealth for VAD, confirmed with a recent federal court decision that the *Criminal Code Act* applies to VAD.^13^ Clinicians are reluctant to use telehealth for VAD due to legal risks. A Commonwealth bill to amend this was introduced in February 2024 and subsequently removed.^14^, ^15^ Advocacy around this problem is ongoing, with current preclusion of electronic communication having significant ramifications for the NT.^16^

## Northern Territory voluntary assisted dying consultation

Between August 2023 and April 2024, the expert advisory panel undertook extensive public consultation to gather Territorians' views on potential VAD legislation and implementation, under the *Inquiries Act 1945* (NT).^17^ As a public consultation process, the process did not require ethics approval, and was overseen by an expert panel, which comprised clinical, legal, consumer, disability and culturally diverse experts, including two Indigenous members. Consultation spanned an online survey with 1 396 responses, 98 written submissions, and public forums, round tables and expert meetings.^3^ The panel prioritised engaging with Aboriginal Territorians and rural and remote stakeholders, developing resources such as plain language guides, posters, radio advertisements, and voice recordings in 14 languages. The final report from the panel is publicly available, and it includes the community member quotations used herein.

### Aboriginal views

Diverse views on VAD were expressed by Aboriginal organisations and individuals.^3^ For example, a community‐controlled organisation stated:

“People might engage better with health services knowing that it could be an option to come back [to country].”^3^

Despite agreement that health services, including VAD, should be accessible to all Territorians, concerns existed about how VAD would be implemented in practice. Potential conflicts between VAD and cultural beliefs were also outlined, including possibilities that some Aboriginal people, particularly elders, were struggling to reconcile VAD with their spiritual, cultural and religious views:

“There will be a division because some people might [not] be able to wrap their head around it … For the older generation, [who have] one foot in the Dreamtime and another foot in religion, this would probably be really difficult.”^3^

Aboriginal decision making also operates within kinship networks,^18^ potentially posing conflicts between Aboriginal collectivist preferences and VAD legislation that mandates individual autonomy free from family coercion. This will require specific mechanisms to accommodate the roles of Aboriginal families in decision making and differing cultural views on illness and death.

There was widespread agreement on the need for cultural safety but also acknowledgement that culture is place‐based and historically contextualised; each person's unique cultural needs must be recognised.^3^ Cultural safety has been defined by Curtis and colleagues,^19^ with elements including health care professionals and organisations examining their own biases, power and cultural influences to provide care deemed safe by patients and their communities. Cultural safety requires ongoing self‐reflection, accountability and action to reduce bias and promote equity.^19^ In this context, risks to cultural safety are associated with power imbalances, medication safety, potential cultural misunderstandings and misconceptions regarding the role of remote PHC services. Similar to other jurisdictions, informed consent must be carefully distinguished from assent.^20^ Co‐design approaches can help address power dynamics, and ensure Aboriginal perspectives are preferenced.^21^, ^22^ Health professionals must prioritise critical self‐reflection to ensure Indigenous world views, customs and values are respected and upheld in end‐of‐life care, including VAD.^19^

Complexities remain regarding representation and authority to speak on behalf of the diverse Aboriginal Nations across the NT. These issues will continue to evolve, highlighting the requirement for iterative consultation with Aboriginal peoples and organisations during VAD implementation.

### Telehealth

Although providing VAD via telehealth is currently illegal, Australia is an international outlier, with New Zealand, the United States, and Canada using telehealth for VAD.^23^, ^24^, ^25^ Some evidence suggests that telehealth can support VAD care and minimise disruption to patients and carers.^25^, ^26^

The NT consultation process revealed diverse views about telehealth. Many participants stressed its importance, raising strong concerns about legal barriers to VAD service delivery in the NT.^3^ Telehealth can improve equity issues around travel, especially for remote patients or those too unwell to travel. One survey respondent stated:

“I think that the difficulties facing rural and remote living people will be enormous so telehealth must be considered.”^3^

Further feedback from the consultation process was that telehealth helped overcome limited availability of medical practitioners outside Darwin and improved access to interpreters, critical for informed consent for VAD. Telehealth would also mitigate infectious disease transmission, such as coronavirus disease 2019.

However, telehealth was also found to have some limitations, including uncertainty about who is present in the room, potentially increasing coercion risks. Health practitioners in another study have expressed that they feel that telehealth is a “second‐rate solution”.^27^ In‐person consultations enable the building of better therapeutic relationships, demonstrating empathy, addressing complex issues surrounding VAD, and interpreting non‐verbal and cultural cues. As one community member stated:

“Doing it over telehealth you'll miss most of the cues. Had a meeting in Groote today regarding court and talking about culturally unsafe practices and the blame that could be put onto the person that's sitting with the judge — took hours to just talk through this. Risks are so significant with telehealth – the follow up could be online if the first appointment is in person.”^3^

For clinicians in remote northern Australia, telehealth has benefits and limitations,^28^ including reducing patient and clinician travel.^3^, ^28^ According to the Royal Australasian College of Physicians (RACP):

“[Telehealth] in the NT in particular, it has played an important role for many years due to well‐known challenges relating to geography, demography, and workforce limitations.”^29^ (Note: this quote relates to telehealth in general. The RACP does not have a position on the intersection of telehealth and VAD).

Mathew and colleagues found that telehealth is more effective with pre‐existing clinician–patient relationships and where patients have familiarity with technology.^28^ Limitations to telehealth included an inadequate clinical and interpreter workforce in remote clinics to support telehealth consultations. Poor internet connectivity, low technology literacy and language barriers have also impacted the use of telehealth. Reliable computer and internet infrastructure can be lacking in remote communities, with patients typically requiring support from clinic staff and interpreters.^28^

### The Northern Territory voluntary assisted dying model of care

The NT has the fewest trained medical practitioners relative to disease burden nationally, managing complex multi‐morbidity, cross‐cultural care and socio‐economic disadvantage.^30^ Adding VAD services will be difficult to accommodate without additional investment.^3^ Telehealth is one possible option, with feedback from Territorians indicating preferences for face‐to‐face consultations, with complementary telehealth access.^3^ Another possibility is that telehealth consultations could be undertaken in the presence of nurses, Aboriginal health practitioners or other appropriate people (such as Aboriginal health workers, community health workers, liaison officers or palliative care support staff), both to mitigate risks and to provide support to patients and families. This is common for other clinical consultations in northern Australia, although baseline telehealth challenges remain.^28^

In the absence of telehealth, other jurisdictions have implemented strategies to support rural and remote patients. For example, Western Australia's VAD Regional Access Support Scheme funds travel for in‐person consultations, reducing disadvantages from geographical barriers and legal telehealth restrictions.^31^ In New South Wales, funded visiting medical officers provide VAD services in certain regional areas.^32^ Equitable access in the NT requires adequate resourcing.

To ensure that cultural issues are adequately addressed, Aboriginal organisations and individuals must remain directly involved in VAD service development and delivery. Co‐design and Aboriginal governance will ensure integration of cultural knowledge and safety, allow continuous adaptation of services based on community feedback and facilitate safe involvement of cultural brokers and interpreters. It will also ensure health care providers receive appropriate cultural competence training on using telehealth for VAD, assuming that telehealth for VAD will become legal in Australia. If it does, safe and voluntary involvement of cultural brokers and interpreters needs to be considered and communication channels with on‐the‐ground health care workers need to be clear.^28^

## Conclusion

Once the nation's leader in VAD, the NT will now be Australia's last jurisdiction to (re)implement voluntary assisted dying. The recent NT consultation process revealed strong preferences for equity of access but also the need to carefully calibrate services to make them culturally safe. This will require additional clinical resources to bring VAD services closer to where patients live, without further overburdening poorly resourced remote PHC services. This will warrant a collaborative approach and expansion of outreach and mobile services. If legalised and with careful attention to cultural safety and clinicians' voices, telehealth could be a viable and safe supplement^28^ to face‐to‐face consultations. Central to the NT context will be Aboriginal input into the governance, design, implementation and iterative evaluation of VAD.

## Competing interests

Geetanjali Lamba and Kane Vellar were part of the Chief Minister's Expert Advisory Panel on voluntary assisted dying. They both receive a salary from NT Health. Some panel work involved travel within the Northern Territory, where travel expenses were covered in line with NT Government policy. No additional salary or payment was taken from the panel work.

## Provenance

Not commissioned; externally peer reviewed.

## Author contributions

Lamba G: Conceptualization, data curation, formal analysis, investigation, methodology, project administration, writing ‐ original draft, writing – review and editing. Vellar K: Conceptualization, investigation, methodology, project administration, writing ‐ original draft, writing ‐ review and editing. Burgess CP: Conceptualization, data curation, supervision, writing ‐ original draft, writing ‐ review and editing. La Brooy C: Conceptualization, supervision, writing ‐ original draft, writing ‐ review and editing. Komesaroff PA: Conceptualization, data curation, supervision, writing ‐ original draft, writing ‐ review and editing.
